# Influence of native and exotic plant diet on the gut microbiome of the Gray's Malayan stick insect, *Lonchodes brevipes*

**DOI:** 10.3389/fmicb.2023.1199187

**Published:** 2023-07-27

**Authors:** Yan Zhen Lim, Yan Hong Poh, Kevin C. Lee, Stephen Brian Pointing, Benjamin J. Wainwright, Eunice Jingmei Tan

**Affiliations:** ^1^Division of Science, Yale-NUS College, Singapore, Singapore; ^2^School of Science, Auckland University of Technology, Auckland, New Zealand; ^3^Department of Biological Sciences, National University of Singapore, Singapore, Singapore

**Keywords:** insect microbiomes, phasmatodea, *Lonchodes brevipes*, host plant, herbivores

## Abstract

Herbivorous insects require an active lignocellulolytic microbiome to process their diet. Stick insects (phasmids) are common in the tropics and display a cosmopolitan host plant feeding preference. The microbiomes of social insects are vertically transmitted to offspring, while for solitary species, such as phasmids, it has been assumed that microbiomes are acquired from their diet. This study reports the characterization of the gut microbiome for the Gray's Malayan stick insect, *Lonchodes brevipes*, reared on native and introduced species of host plants and compared to the microbiome of the host plant and surrounding soil to gain insight into possible sources of recruitment. Clear differences in the gut microbiome occurred between insects fed on native and exotic plant diets, and the native diet displayed a more species-rich fungal microbiome. While the findings suggest that phasmids may be capable of adapting their gut microbiome to changing diets, it is uncertain whether this may lead to any change in dietary efficiency or organismal fitness. Further insight in this regard may assist conservation and management decision-making.

## 1. Introduction

Herbivorous insects can host microorganisms, such as bacteria, viruses, fungi, archaea, and protozoa in their digestive tract, referred to as their gut microbiome (Gurung et al., 2019; Muñoz-Benavent et al., 2021). The gut microbiome of insects has been implicated in the transformation of food substrates and acting as a direct dietary source providing essential nutrients lacking in the plant diet (Dillon and Dillon, 2003; Moran et al., 2019). Evidence for an adapted lignocellulosic microbiome is strong for some insect groups, notably termites (König et al., 2013; Jang and Kikuchi, 2020). Studies have shown that bacteria and fungi in insect guts possess enzymes breaking down insoluble plant polymers, including cellulose and hemicellulose, in addition to soluble compounds such as starch and pectin (Watanabe and Tokuda, 2010; Jang and Kikuchi, 2020). Additional roles for the gut microbiome have been inferred from studies of other animal systems and include protection against pathogen infection (Mendes and Raaijmakers, 2015; Raymann and Moran, 2018; Jang and Kikuchi, 2020) and behavioral influences (Davidson et al., 2018, 2020; Jones et al., 2018).

The recruitment of gut microbiomes in insects is not well constrained, with studies on social insects such as bees and termites suggesting direct transfer between individuals [e.g., (Koch and Schmid-Hempel, 2011; Bourguignon et al., 2018)], while other studies indicate that gut microbiomes may reflect recruitment from dietary substrates (Jones et al., 2019). Studies on lepidopterans, for instance, demonstrated a correlation between diet and gut microbial diversity, where the host plants were the main contributors to the herbivorous insects' gut microbiome (Mason et al., 2020). The extent of the effect of diet on gut microbiome differed depending on both the lepidopteran host and host plant species (Jones et al., 2019) and their exposure to environments with soil (Hannula et al., 2019). In herbivorous insects, the gut microbiome has been shown to largely reflect the host insect phylogeny rather than their ecology (McLean et al., 2019). The extent to which the insect gut acts as an environmental filter that shapes the diversity of its microbiome toward taxa that are beneficial symbionts for the insect host appears to depend upon the insect taxa (Moran et al., 2019; Mason, 2020).

Phasmids (stick insects) are non-social folivorous insects that feed almost exclusively on leaves but are able to adapt to foreign diets, that is, plant species that the insects are not usually exposed to or would consume, if the favored host is unavailable (Boucher and Varady-Szabo, 2005; Blüthgen et al., 2006; Shelomi et al., 2014a). The current understanding of gut microbiomes in phasmids is not well constrained. In one study, bacterial gut microbiomes showed no significant differences in composition across several phasmid families (Shelomi et al., 2014a). By sequencing the gut contents, one study found that Proteobacteria was dominant, followed by Actinobacteria and Firmicutes, and *Spiroplasma* spp. and *Sphingobium* spp. were the most common microbes isolated (Shelomi et al., 2013). In another study, cultured bacterial strains from phasmid guts affiliated with the Actinomycetales, Enterobacteriales, Lactobacillales, Pseudomonadales, and Xanthomonadales (Shelomi et al., 2015). It has been suggested that the environment and diet can affect the gut microbiome of phasmids (Ignasiak and Maxwell, 2017). Furthermore, gut microorganisms found in phasmids were shown to produce cell wall degrading enzymes including cellulases important to the digestion of plant matter (Shelomi et al., 2014b), and it has also been postulated that some phasmids possess their own genes for breakdown of soluble plant carbohydrates, for example, pectin (Shelomi et al., 2016). For species of phasmids that do not adhere their eggs to the leaf substrate, this stage in life history is unlikely to be a route for microbiome recruitment from the plant substrate.

Here, we set out to further interrogate the hypothesis that different plant diets in phasmids would lead to shifts in the taxonomic composition of the gut microbiome. This is of relevance to Singapore because of the high levels of urbanization with a mixture of native and exotic plant host species and the potential for a shift in plant hosts of herbivores. Specifically, we sought to test the following hypotheses: (a) Does phasmid gut microbiota vary with diet, and (b) is phasmid gut microbiota a subset of food (i.e., plant) or local soil microbiomes? We reared a local species, Gray's Malayan stick insect, *Lonchodes brevipes*, on two species of host plants. This phasmid is encountered in mangroves and rainforests (Seow-Choen, pers. obs.) and feeds on sea hibiscus (SH), *Hibiscus tiliaceus* in the wild, and does not adhere their eggs to the leaf substrate. In hobbyist cultures, this phasmid is commonly reared on a widespread non-native ornamental plant, golden penda (GP), *Xanthostemon chrysanthus*. We compared the gut microbiome of individuals reared on both plants, as well as the microbiome of the host plant leaves and soil because soil is the most microbially emissive component of the habitat.

## 2. Materials and methods

### 2.1. Experimental subjects and environmental samples

We used laboratory-reared offspring from field-caught *Lonchodes brevipes* in this study. This ensured that no adaptations to domestication were introduced. Phasmids were housed individually in a temperature-controlled laboratory at 24°C in a 12-h:12-h light: dark cycle. All individuals were provided food plants *ad libitum* and monitored to be feeding and behaving normally prior to the trials. Individuals with missing or misshapen legs or body parts that may have affected their feeding behavior were not used in our experiments. We used a total of 20 nymphs between 12 and 16 weeks of age; 10 nymphs were raised since hatching on the leaves of the golden penda while another 10 nymphs were raised on sea hibiscus. From initial tests, frass collected from single days yielded insufficient DNA; therefore, frass was collected from individual nymphs daily over 7-day intervals and pooled for DNA extraction. In total, 10 samples of each of the host plant leaves and topsoil within a meter around the tree trunks were collected aseptically into sterile containers. As the vegetation on campus is regularly sprayed with or exposed to pesticides, all leaves were washed prior to feeding the phasmids. Therefore, for consistency, leaves used for sequencing were also washed prior to DNA extraction.

### 2.2. DNA extraction, PCR amplification, and sequencing

First, leaf and frass samples were flash frozen in liquid nitrogen and ground with the mortar and pestle. We extracted the DNA from leaf samples using FavorPrep^TM^ Plant Genomic DNA Extraction Mini Kit (Favorgen), while DNA extraction from frass and soil samples was performed using QIAamp PowerFecal Pro DNA Kit (QIAGEN). To maximize yield, phenol:chloroform:isoamyl alcohol was added to the frass prior to homogenization. Extracted DNA samples were then cleaned with the DNeasy PowerClean Pro Cleanup Kit (QIAGEN).

PCR amplification of the V4 region of the 16S SSU rRNA gene was performed using the bacterial and archaeal primers 515F and 806R [515F—GTG CCA GCM GCC GCG GTA A; 806R—GGA CTA CHV GGG TWT CTA AT; Caporaso (2011)]. Forward and reverse primers were modified to include Illumina adapters, a linker, and a unique barcode (Caporaso, 2011). Each reaction was performed in a total volume of 25 μl, containing 0.75 μl of each primer at 10 μM, 12.5 μl of buffer, 1.5 μl BSA at 10 mg/ml, 0.5 μl of MgCl_2_, 0.1 μl of enzyme (KAPA 3G, Kapa Biosystems, Inc, Wilmington, MA, USA), 1 μl of undiluted DNA, with H_2_O added to make up to 25 μl. PCR cycling condition was set at 94°C for 180 s, followed by 35 cycles of 94°C for 45 s, 50°C for 60 s, and 72°C for 90 s, and a final extension at 72°C for 10 min. Negative PCR controls were included to identify any potential laboratory reagent contamination.

Fungal DNA amplification was performed with the ITS1F [5′-CTT GGT CAT TTA GAG GAA GTA A-3′; Gardes and Bruns (1993)] and ITS2 [5′-GCT GCG TTC TTC ATC GAT GC-3′; White et al. (1990)] primer set. Primers were modified to include a unique barcode, linker, and Illumina adapter [see Smith and Peay (2014) for exact details]. Each reaction was performed in a total volume of 25 μl, containing 0.75 μl of each primer at 10 μM, 12.5 μl of buffer, 1.5 μl BSA at 10 mg/ml, 0.5 μl of MgCl_2_, 0.1 μl of enzyme (KAPA 3G, Kapa Biosystems, Inc, Wilmington, MA, USA), 1 μl of undiluted DNA, with H_2_O added to make up to 25 μl. PCR cycling condition was set at 95°C for 3 min, followed by 35 cycles of 95°C for 20 s, 53°C for 15 s, and 72°C for 20 s with a final extension at 72°C for 60 s. Negative PCR controls substituting water for DNA were included to aid in the detection of contamination.

Successful PCR amplification was confirmed on a 1% TAE buffer agarose gel. normalization and cleaning of PCR products were performed in sequalprep normalization plates (Invitrogen, Frederick, 129 Maryland, USA). Both libraries were sequenced independently on the Illumina MiSeq platform (600 130 cycles, V3 chemistry, 300-bp paired end reads) with a 30% PhiX spike (Macrogen, Korea).

### 2.3. Data analysis

The amplicon sequencing data were processed using the R package dada2 v.1.18.0 (Callahan et al., 2016) to infer distinct amplicon sequence variants (ASVs). The workflow was as follows: primer sequences were first removed via cutadapt v3.3 (Martin, 2011). The bacterial reads were uniformly trimmed to the length of 230/220 bp (forward/reverse) and filtered to remove reads exceeding 2/5 (forward/reverse) expected errors or reads shorter than 175 bp. The fungal reads were not trimmed, but reads exceeding 5/8 bp (forward/reverse) expected errors or reads shorter than 50 bp were removed. In both cases, any reads with N or those identified as possible PhiX sequences were removed. The ASVs were inferred with pseudo-pooling to increase sensitivity and preserve singletons for richness estimations. Potential chimeric sequences were removed, and the inferred ASVs were classified using the naive Bayesian classifier implemented in dada2 with the reference database UNITE v8.2 eukaryote (Nilsson et al., 2018) for fungi and SILVA v138 (Quast et al., 2013) for bacteria. The R package phyloseq v1.36.0 (McMurdie and Holmes, 2013) was used to handle the resulting ASV abundance table for ecological analyses and visualizations. The R package decontam v1.12.0 (Davis et al., 2018) was used to identify putative contaminant ASVs by removing ASVs identified by either the frequency (threshold = 0.1) or the prevalence (threshold = 0.5) method, based on the taxa in the control samples and the DNA concentrations of the initial extractants (Nanodrop, Thermo Fisher Scientific, USA). After removing ASVs associated with non-bacterial or non-fungal (genomic) sources (e.g., mitochondria and chloroplast), samples with < 1,000 reads were removed due to insufficient information and potential bias that they may introduce. Excluding control samples, the processing and filtering resulted in 69 fungi samples with a total of 3,417,410 counts over 3,452 ASVs, as well as 72 bacterial samples with a total of 2,683,111 counts over 8,314 ASVs. The taxonomy and taxa named are based on the SILVA reference database that we used.

General processing of the community data including the calculation of relative abundance and estimates of alpha diversity was conducted using the R package phyloseq (McMurdie and Holmes, 2013) and visualized using ggplot2 (Wickham, 2016). The proportional differences of bacterial and fungal families in the samples belonging to the two categories (host plant) were compared using the R package ANCOM-BC v.1.2.2; the differences (enriched in one group vs. another) were expressed as the *W* statistic, which reflects the effect size. A larger positive *W* indicates enrichment in sea hibiscus, and a more negative W indicates enrichment in golden penda. Significant differences were based on *p*-values adjusted by the Holm–Bonferroni method (adj. *p* < 0.05). Therefore, the two extremes (positive or negative *W*) indicated the largest differences between the two groups of frass samples. Significance testing of the community structure in relation to sample type and the associated host plant species was conducted using “manyglm,” with a negative binomial distribution, from the R package mvabund v.4.2.1 (Wang et al., 2012).

## 3. Results and discussion

**Figure 1:**
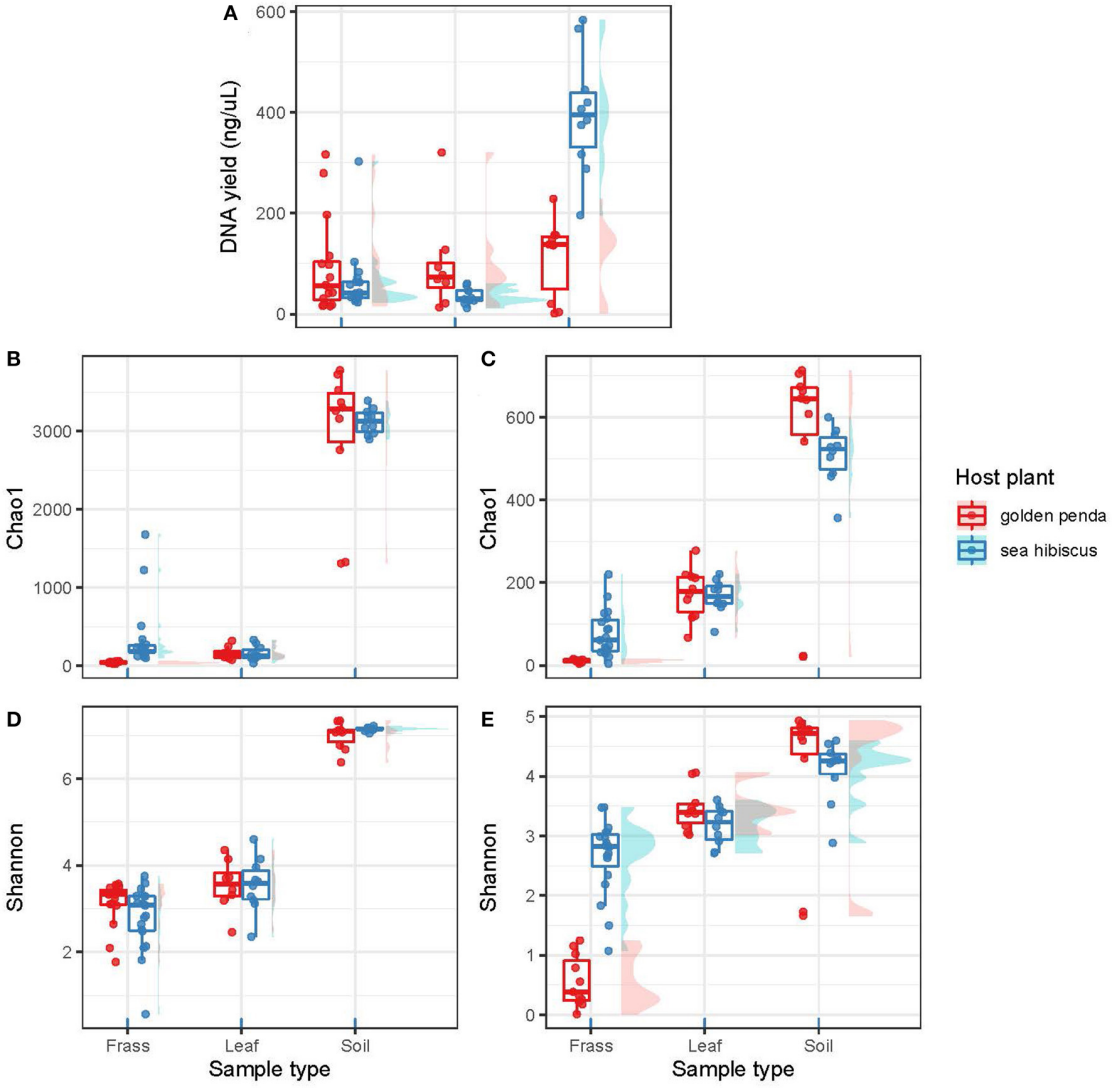
The plots are organized into two columns with the bacterial assemblages on the left and the fungal assemblages on the right. **(A)** shows DNA yields from the various substrates (frass, leaf, soil). **(B, C)** show taxonomic richness using the Chao1 richness estimator. **(D, E)** show taxonomic diversity as Shannon's index (H′). Each data point in a box plot represents a biological sample. The data points are grouped into sample types (frass, leaf, and soil) and further separated by associated host plant species.

The high-throughput sequencing of bacterial and fungal diversity in phasmid frass was used as a proxy for gut microbiomes. This approach is congruent with that adopted for most other studies of microbiomes in animal and human systems, for example, Huttenhower et al. (2012). We used this approach to interrogate the central hypothesis that native and introduced plant food sources impacted the composition of phasmid gut microbiomes. Comparison of microbiota associated with food sources and surrounding soils provided triangulation of potential sources and influence on the composition of the gut microorganisms. The DNA yield from samples was used as a general proxy for overall (i.e., bacterial and fungal) microbial biomass, although we recognize that host DNA may also have been a confounding factor. This indicated that while the food substrate and frass had similar levels of biomass (Figure 1A), the soil samples yielded significantly greater microbial biomass per gram of substrate than frass (*p* = 0.001) and leaves (*p* < 0.001). Of note was the markedly higher biomass in soil associated with native vs. exotic plants, and this may indicate a more developed rhizosphere microbiome for the native plants that reflects their natural history.

Contrasting alpha diversity estimates were observed for bacteria and fungi across the sample types. Bacterial richness estimates (Chao1) were significantly higher in the frass of phasmids fed on native (sea hibiscus) vs. exotic (golden penda) leaf substrates (Figure 1B; Mann–Whitney *U* = 0, *n*_1_ = 16, *n*_2_ = 19, *p* < 0.001). Similarly, fungal richness was significantly higher in the frass of phasmids reared on native vs. exotic leaves (Figure 1C; Mann–Whitney *U* = 0, *n*_1_ = 16, *n*_2_ = 19, *p* < 0.001). The Shannon's index estimates that consider richness and evenness were not significantly different for bacteria between native and exotic substrates (Figure 1D), whereas for fungi estimates, richness and evenness were significantly higher for the native substrate (Figure 1E; Mann–Whitney *U* = 2, *n*_1_ = 11, *n*_2_ = 19, *p* < 0.001). The leaves of native and exotic plants displayed similar bacterial richness to that of frass while the soil as expected supported a markedly greater diversity of bacteria (Thompson et al., 2017). In contrast, both leaves and soil supported greater fungal richness than frass, and this reflected the importance of the phyllosphere as a reservoir of fungal diversity (Xu et al., 2022).

**Figure 2:**
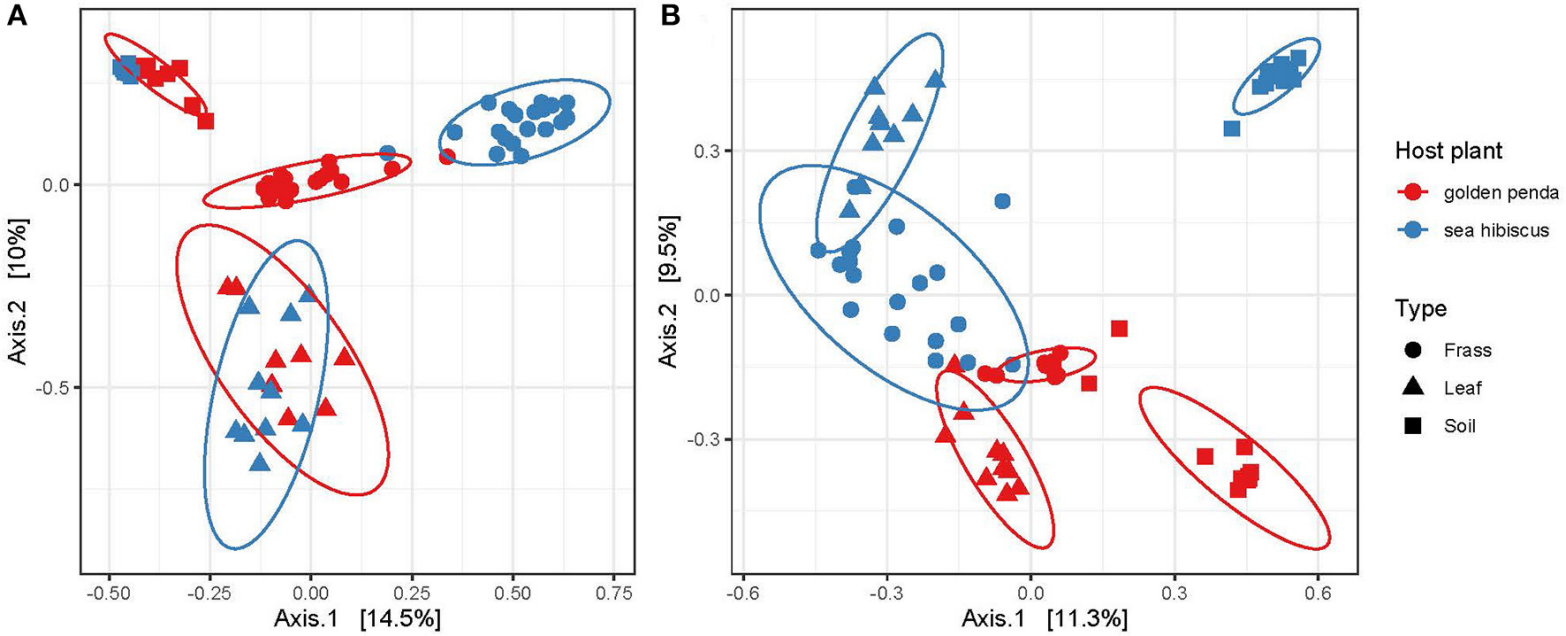
Ordination of similarities between **(A)** bacterial and **(B)** fungal communities via principal coordinate analysis of Hellinger distances of the rarefied ASVs. Each assemblage derived from a sample is shown as a data point, with the sample type indicated by its shape and the associated host plant by its color. The clustering of each group is highlighted by a 95% confidence interval ellipse assuming a multivariate t-distribution.

Beta diversity patterns measured by Hellinger distances revealed that gut bacterial assemblages were more similar to each other than their associated leaf or soil assemblages regardless of the leaf diet, suggesting that bacteria in the gut had undergone environmental filtering and recruitment (Figure 2A). In contrast, the fungal assemblages in frass from the different diets displayed less overlap with each other but more overlap with the corresponding leaf substrate, thus suggesting recruitment from the leaf substrate may be important for fungi (Figure 2B). For both bacteria and fungi, the frass assemblages displayed no overlap in overall similarity with soil assemblages. This raises the interesting question of what the recruitment sources are for gut bacteria and fungi in phasmids. The leaves and soil were unable to fully explain the observed diversity in frass and so additional sources might include vertical transmission by phasmids, or other aeolian or environmental sources. Interestingly, the results also revealed that bacteria and fungi in soil respond differently to native and exotic plants, with bacteria assemblages remaining essentially unchanged while a significant shift in fungal assemblages occurred in soils planted with native vs. exotic plants (Figures 2A, B). The altered soil mycobiome with exotic plants may thus have undetermined impacts on the functioning of the soil and phyllosphere. This has potential implications for horticultural management and conservation.

**Figure 3:**
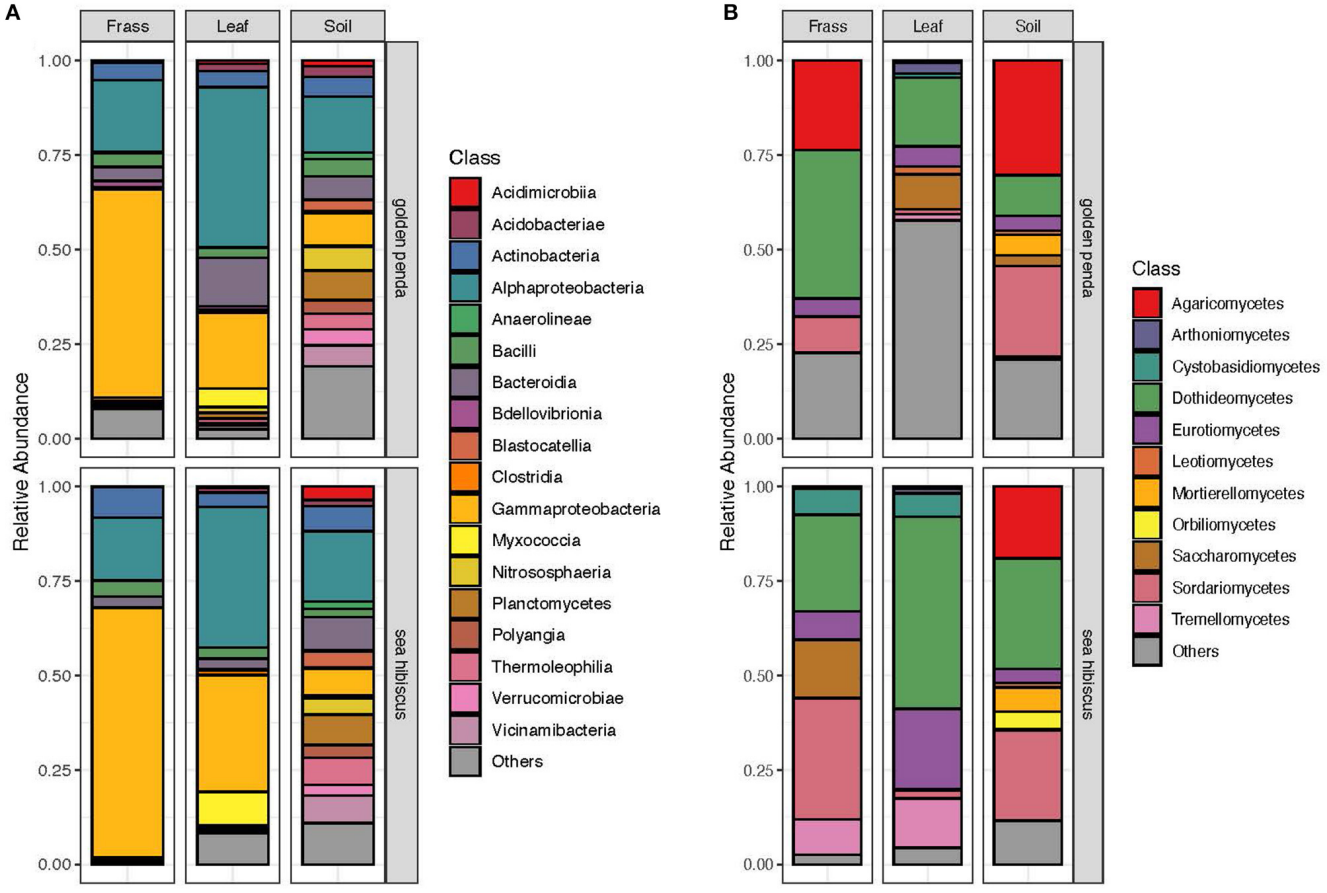
Overall community composition of **(A)** bacteria and **(B)** fungi samples at the class level, by sample type and source plant species, and expressed as relative abundances in each of the groups. Classes with mean relative abundance of <0.5% are pooled into the “Others” category.

**Figure 4:**
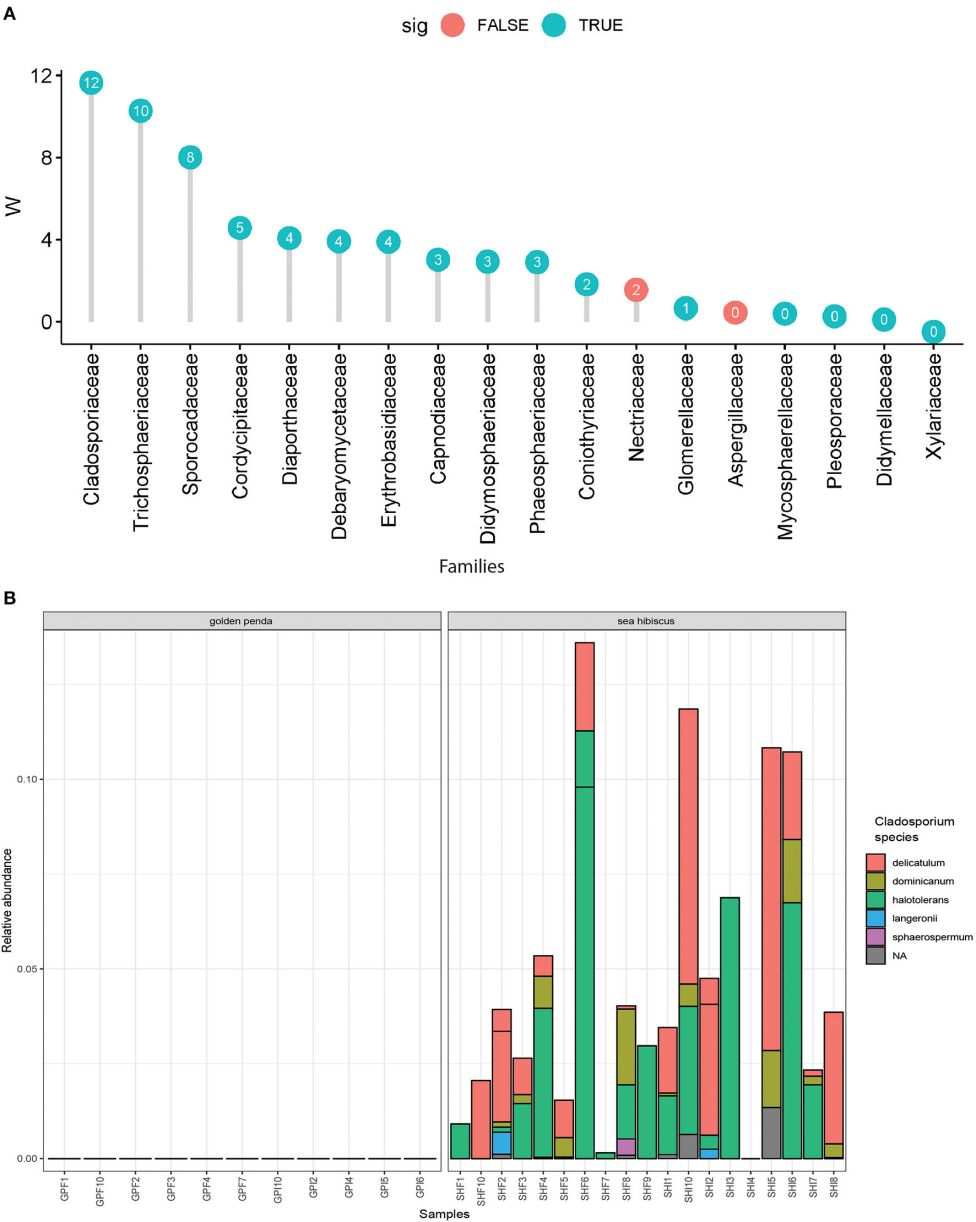
Differential abundance analysis of fungi. **(A)** Overall dominant fungal families in frass samples that were over >1% mean relative abundance. Effect sizes where W = >0 indicated the family was overall more abundant in frass from sea hibiscus leaf substrate, whereas none were more abundant in frass from golden penda leaf substrate (i.e., W = < 0). A teal-colored data point indicates the difference in abundance of the family is statistically significant (adj. *p* < 0.05), while a red-colored data point indicates the association was not supported. **(B)** Species within the fungal genus *Cladosporium* were strongly associated with frass produced from sea hibiscus and virtually absent in the golden penda frass.

**Figure 5:**
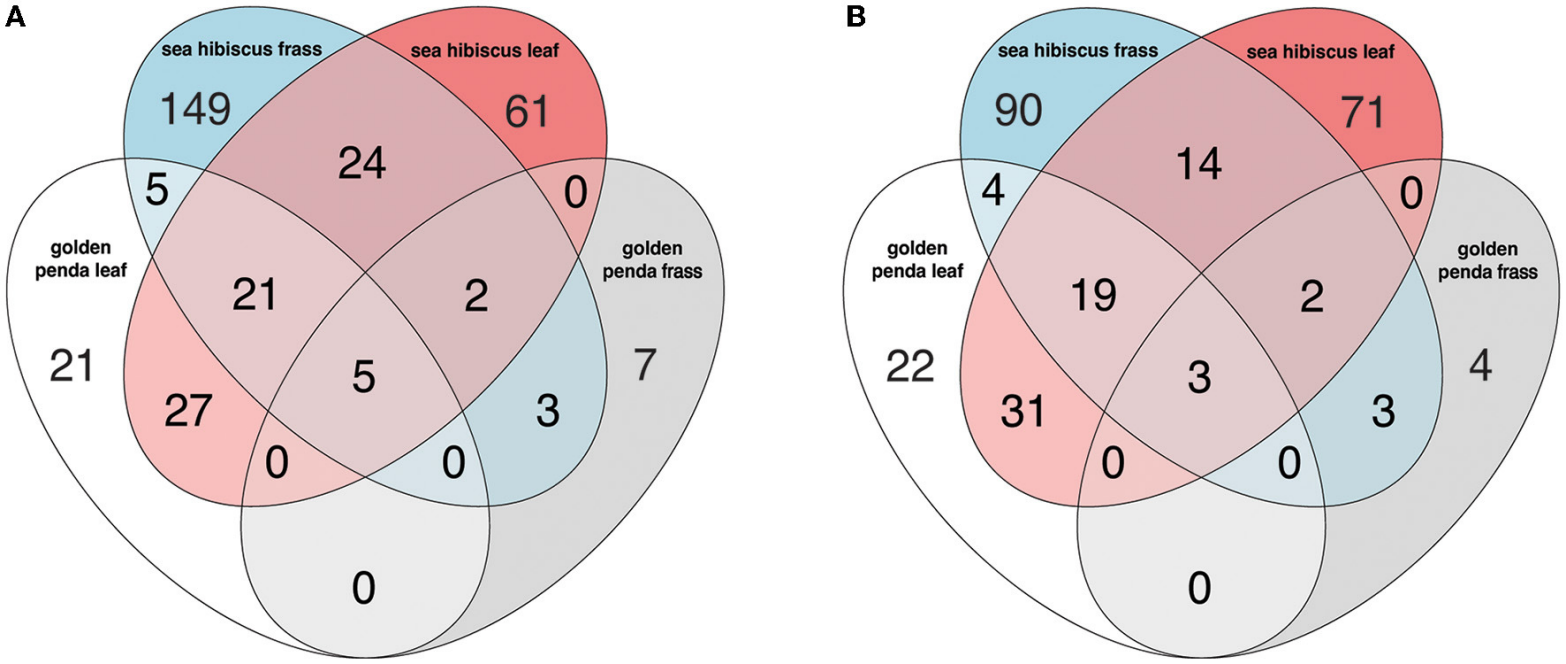
The number of core ASVs shared between groups based on sample type (frass, leaf, soil) and associated host plant (golden penda or sea hibiscus) in the **(A)** bacteria and **(B)** fungi assemblages. A core ASV for a group of samples is here defined as being above 0.1% in relative abundance in over 25% of samples.

Taxonomic diversity estimation revealed that frass bacterial assemblages were enriched in taxa within Alpha- and Gamma-proteobacteria. The most frequently encountered bacterial families were Pseudomonadaceae and Enterobacteriaceae for the native plant-fed insects and Comamonadaceae and Enterobacteriaceae in the exotic plant-fed insects (Figure 3A; Supplementary Figure 1). Interestingly, while Enterobacteriaceae is abundant in both groups of frass samples, in SH, the family consisted almost entirely of the genus *Klebsiella*, whereas in GP, *Salmonella* was also present. Patterns in diversity for fungi were less evident (Figure 3B; Supplementary Figure 2) due to the high taxonomic diversity and large numbers of unclassified taxa. Many of the fungal genera (e.g., *Aspergillus* and *Penicillium*) include known cellulose-degrading taxa (Vázquez-Montoya et al., 2020), although whether genetic signals represent vegetative cells rather than dormant spores requires these data to be interpreted with some caution. In order to further interrogate the likelihood that gut microbiomes of insects reared on native and exotic plant feed had distinct assembly patterns, we conducted differential abundance analysis to identify bacterial and fungal families that differed significantly (i.e., were enriched) between frass samples from phasmids fed on the two plant hosts (Figure 4A). Notably, the strong differential abundance pattern for *Cladosporium* species (enriched in SH frass and virtually absent in GP frass) may indicate the effect of selection pressure due to the gut environment affected by the different diets (Figure 4B). The core ASVs shared between the sample type (frass, leaf, or soil) and associated host plant (golden penda or sea hibiscus) varied greatly, with the greatest number of core ASVs shared between the leaves of the two host plant species (Figure 5), while the majority of core ASVs were only found in the respective host plant leaves and frass of insects feeding on sea hibiscus, that is, not shared between communities in different sample types (Figure 5).

Overall, this study shows that community structure was driven by host selection instead of dispersal from the environment, as the environments (soil/leaf) were not as different as the frass associated with two source plant species. Nonetheless, the sample types (i.e., frass, leaf, and soil), the associated host plant species, and the interaction term of the two factors were all statistically significant in structuring the bacterial and fungal communities observed in this study (manyglm, all *p* < 0.001). Furthermore, the microbiomes varied significantly in response to a native or exotic plant diet, with fungi accounting for more variation than bacteria. While the gut communities of some herbivorous insects are characterized by transient and food-derived bacteria (Whitaker et al., 2016; Hernández-García et al., 2017), other studies do not find a strong correlation between diet and the gut microbiome (Mason and Raffa, 2014; Chaturvedi et al., 2017). A previous study suggests that phasmids are well adapted to changes in diets and may not be reliant on their gut microbiome for specialized digestion of plants (Shelomi et al., 2014b). Our study provides additional insight that indicates a shift in diet, resulting in measurable changes to the insect gut microbiome that includes known cellulolytic taxa. The data also indicate that exotic plants result in altered soil and herbivorous insect microbiomes and this is relevant to the conservation and management issues where factors that potentially affect insect herbivory are important (Bahrndorff et al., 2016). Finally, the characterization of the *Lonchodes brevipes* microbiome also adds to the growing global inventory of animal microbiomes (Douglas, 2019).

## Data availability statement

The datasets presented in this study can be found in online repositories. The names of the repository/repositories and accession number(s) can be found in the article/Supplementary material.

## Author contributions

ET and SP conceived the project, interpreted the findings, and wrote the manuscript. YL and YP performed the specimen collections and DNA extractions. BW performed PCR amplification and sequencing. KL processed the sequencing data. All authors read and agreed on the final manuscript.

## Supplementary material

Supplementary Figure 1 Distribution of bacteria in samples sorted according to PCoA1 (45.1%). **(A)** Relative abundance of bacterial families; **(B, C)** Relative abundance of bacterial genera for the two host plants.

Supplementary Figure 2 Distribution of fungal samples sorted according to PCoA1 (21.6%). **(A)** Relative abundance of fungal families; **(B, C)** Relative abundance of fungal genera for the two host plants.
