# Supplementary figures and images for: Influence of native and exotic plant diet on the gut microbiome of the Gray's Malayan stick insect, Lonchodes brevipes
Source: Front Microbiol. 2023 Jul 27;14:1199187. doi: 10.3389/fmicb.2023.1199187 (PMC10412900; doi:10.3389/fmicb.2023.1199187)

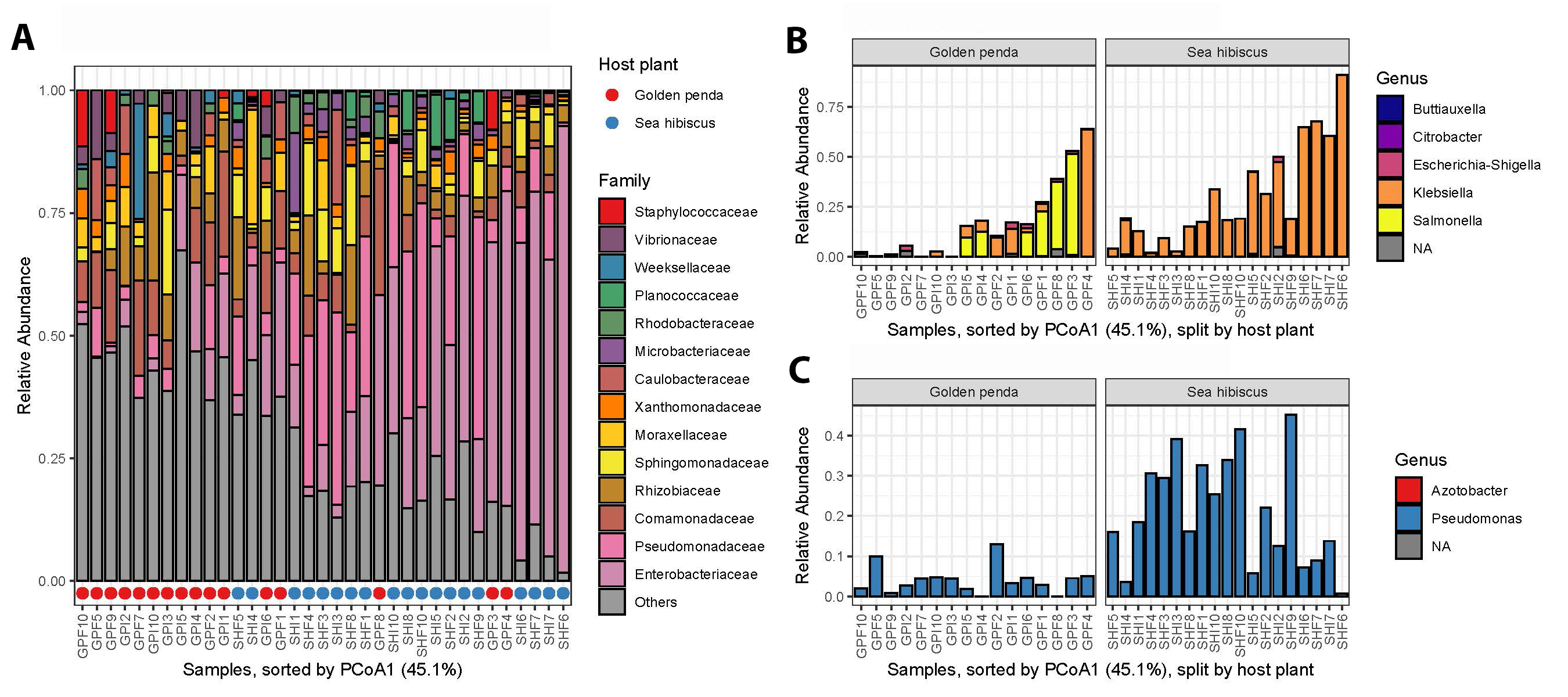

Supplement: Supplementary Figure 1 — Distribution of bacteria in samples sorted according to PCoA1 (45.1%). (A) Relative abundance of bacterial families; (B, C) Relative abundance of bacterial genera for the two host plants. [file Image_1.tif]

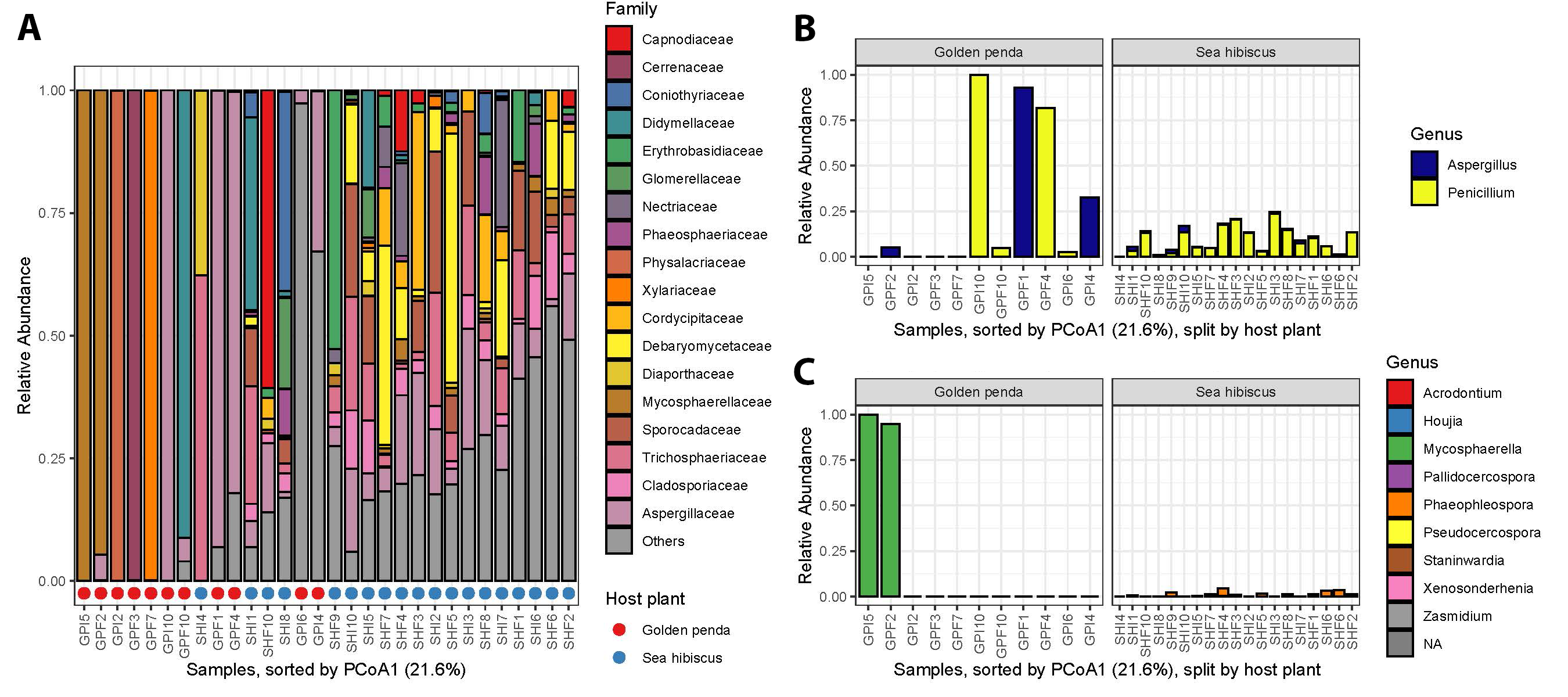

Supplement: Supplementary Figure 2 — Distribution of fungal samples sorted according to PCoA1 (21.6%). (A) Relative abundance of fungal families; (B, C) Relative abundance of fungal genera for the two host plants. [file Image_2.tif]
